# The association between monocyte to high-density lipoprotein ratio and hemorrhagic transformation in patients with acute ischemic stroke

**DOI:** 10.18632/aging.102757

**Published:** 2020-02-05

**Authors:** Yanan Wang, Yajun Cheng, Quhong Song, Chenchen Wei, Junfeng Liu, Bo Wu, Ming Liu

**Affiliations:** 1 Department of Neurology, West China Hospital, Sichuan University, Chengdu 610041, Sichuan, China

**Keywords:** monocyte, high-density lipoprotein, hemorrhagic transformation, ischemic stroke

## Abstract

Hemorrhagic transformation (HT) is a common complication in patients with acute ischemic stroke. We investigated whether the monocyte to high-density lipoprotein ratio (MHR) is related to HT. Consecutive patients with ischemic stroke within 24 h of symptom onset were included in this study. HT was diagnosed by follow-up brain imaging after admission, and was classified as asymptomatic or symptomatic according to whether patients showed any neurologic worsening. Logistic regression was performed to estimate the association between MHR and HT. Of the 974 enrolled patients, 148 (15.2%) developed HT, and 24 (2.5%) patients experienced symptomatic HT. Compared to the highest MHR tertile (> 0.37), the lowest MHR tertile (< 0.22) was associated with 1.81-fold increase (95% CI 1.08-3.01, *P* = 0.024) in the odds of HT and 3.82-fold increase (95% CI 1.04-14.00, *P* = 0.043) in the odds of symptomatic HT after adjustment for possible confounders. Using a multivariate logistic regression model with restricted cubic spline, we found that elevated MHR was associated with a decreased risk of HT and symptomatic HT. In summary, lower MHR was independently associated with increased risk of HT and symptomatic HT in patients with ischemic stroke.

## INTRODUCTION

Hemorrhagic transformation (HT) is a common complication in patients with acute ischemic stroke [1]. The rate of HT ranges from 7.5% to 49.5% [2–5], whereas the incidence of symptomatic HT ranges from 0.6% to 20% [6], depending on whether reperfusion therapy is performed. HT, especially symptomatic HT, is associated with poor prognosis and possibly influences the clinical decision- making on usage of thrombolytic and anticoagulant agents [7]. Therefore, it is necessary to understand the underlying mechanism of HT and identify associated risk factors, which may help clinicians select appropriate treatments and improve patients’ outcome.

Growing evidence suggests that HT is facilitated by post-stroke inflammatory response, and its impact on blood brain barrier [8]. Monocytes and macrophages are essential components in innate immunity and can modulate both the pro-inflammatory and anti-inflammatory process [9]. A clinical study reported that a subpopulation of monocytes is beneficial with a phenotype that is associated with lower mortality after ischemic stroke [10]. A preclinical study showed that monocytes/macrophages may prevent HT in mice [11]. However, whether monocytes are related to HT in humans remains unclear. Likewise, there have been conflicting findings about the association between high-density lipoprotein (HDL) cholesterol and HT. Several studies indicated that high levels of HDL increased the risk of HT [12, 13], while others observed no association [14]. Recently, the monocyte to HDL cholesterol ratio (MHR), which combines pro-inflammatory and anti-inflammatory process, is found to be a novel prognostic marker of cardiovascular diseases [15], immune system diseases [16] and rheumatic disease [17]. However, evidence on the association between MHR and HT is limited.

In the present study, we aimed to investigate the association between MHR and HT in patients with ischemic stroke.

## RESULTS

### Baseline characteristics

During the study period, 1035 patients with ischemic stroke patients met the study criteria, of whom 61 (5.9%) were excluded: 25 patients had HT on admission, 32 patients did not undergo a second computed tomography (CT) or magnetic resonance imaging (MRI) scan and 4 patients lacked data on serum monocyte or HDL within 24 h of hospital admission. Finally, 974 patients were included in this study. The median age was 69 years (interquartile ranges [IQR]: 58–78) and 573 (58.8%) subjects were males. Baseline characteristics are presented in Table 1.

A total of 148 (15.2%) patients developed HT and 24 (2.5%) patients experienced symptomatic HT. In comparison to patents without HT, those with HT were significantly older (70.7 ± 12.5 vs. 66.8 ± 13.8, *P* = 0.001) and contained a significantly smaller proportion of males (45.9% vs. 61.1%, *P* < 0.001). HT group comprised a higher frequency of atrial fibrillation (30.4% vs. 14.0%, *P* < 0.001) and large infarct size (60.8% vs. 15.9%, *P* < 0.001) as well as significantly higher NIHSS score on admission (14 vs. 5, *P* < 0.001). In addition, patients with HT had lower values of systolic blood pressure (143 ± 23 vs. 148 ± 24 mmHg, *P* = 0.009), monocyte count (0.32 vs. 0.36, *P* = 0.016), triglyceride (1.09 vs 1.34 mmol/L, *P* < 0.001) and low-density lipoprotein cholesterol (2.46 vs. 2.54 mmol/L, *P* = 0.032), but had higher HDL (1.32 vs. 1.22 mmol/L, *P* = 0.008). HT group was less likely to receive antiplatelets (83.8% vs. 93.0%, *P* < 0.001) and lipid-lowering agents (86.5% vs. 93.2%, *P* = 0.005), but more likely to receive thrombolysis (16.2% vs. 9.0%, *P* = 0.007) and thrombectomy (15.5% vs. 6.4%, *P* < 0.001).

### Association between MHR and HT

MHR in healthy controls was significantly lower than that in ischemic stroke patients (0.26 vs. 0.29, *P* < 0.001). Moreover, patients with HT had a significantly lower MHR compared to those without HT (0.25 vs. 0.30, *P* < 0.001; Table 1). Similar results were observed when patients were categorized by symptomatic HT or not (0.23 vs. 0.29, *P* < 0.001; Table 1).

**Table 1:** Baseline characteristics of included patients according to the subcategorized groups of hemorrhagic transformation.

	**No HT (n=826)**	**HT (n=148)**	***P*-value**	**No symptomatic HT (n=950)**	**Symptomatic HT (n=24)**	***P*-value**
Demographics						
Age (years)	66.8 ± 13.8	70.7 (12.5)	0.001	67 ± 14	74 ± 12	0.012
Male, n (%)	505 (61.1)	68 (45.9)	<0.001	560 (58.9)	13 (54.2)	0.678
Medical history						
Hypertension, n (%)	446 (54.0)	81 (54.7)	0.869	513 (54.0)	14 (58.3)	0.674
Diabetes mellitus, n (%)	176 (21.3)	30 (20.3)	0.776	201 (21.2)	5 (20.8)	1
Hyperlipidemia, n (%)	28 (3.4)	3 (2.0)	0.609	30 (3.2)	1 (4.2)	0.544
Atrial fibrillation, n (%)	116 (14.0)	45 (30.4)	<0.001	152 (16.0)	9 (37.5)	0.010
Previous medication use						
Antiplatelets, n (%)	96 (11.6)	15 (10.1)	0.6	109 (11.5)	2 (8.3)	0.675
Lipid–lowering agents, n (%)	56 (6.8)	10 (6.8)	0.992	64 (6.7)	2 (8.3)	0.675
Anticoagulants, n (%)	45 (5.4)	7 (4.7)	0.844	51 (5.4)	1 (4.2)	1
Current smoking, n (%)	326 (39.5)	49 (33.1)	0.143	368 (38.7)	7 (29.2)	0.401
Current drinking, n (%)	206 (24.9)	27 (18.2)	0.079	226 (23.8)	7 (29.2)	0.627
Clinical features						
NIHSS on admission, median (IQR)	5 (2–12)	14 (9–20)	<0.001	6 (3–13)	13 (10–18)	<0.001
SBP (mmHg)	148 ± 24	143 ± 23	0.009	147 ± 24	155 ± 29	0.128
DBP (mmHg)	85 ± 15	85 ± 18	0.61	85 ± 15	91 ± 24	0.077
Glucose (mmol/L)	7.98 ± 3.28	8.21 ± 2.39	0.412	8.0 ± 3.2	8.4 ± 2.7	0.561
WBC count (× 109/L), median (IQR)	7.39 (5.99 – 9.3)	8.2 (6.5–9.5)	0.044	7.49 (6.10 – 9.36)	7.01 (5.30 – 8.73)	0.132
Monocyte count (× 109/L), median (IQR)	0.36 (0.27 – 0.47)	0.32 (0.24 – 0.44)	0.016	0.36 (0.26 – 0.47)	0.30 (0.25 – 0.34)	0.015
TG (mmol/L), median (IQR)	1.34 (0.92 – 1.97)	1.09 (0.77 – 1.48)	<0.001	1.29 (0.90 – 1.91)	0.98 (0.76 – 1.84)	0.162
TC (mmol/L), median (IQR)	4.31 (3.66 – 5.07)	4.21 (3.47 – 4.70)	0.053	4.30 (3.64 – 5.02)	4.00 (3.31 – 4.57)	0.106
HDL (mmol/L), median (IQR)	1.22 (0.99 – 1.47)	1.32 (1.06 – 1.54)	0.008	1.23 (0.99 – 1.48)	1.36 (1.09 – 1.61)	0.185
LDL (mmol/L), median (IQR)	2.54 (1.98 – 3.23)	2.46 (1.90 – 2.96)	0.032	2.52 (1.97 – 3.20)	2.26 (1.68 – 2.93)	0.108
MHR, median (IQR)	0.30 (0.20–0.43)	0.25 (0.17–0.38)	0.001	0.29 (0.20–0.42)	0.23 (0.17–0.30)	0.015
Large infarct size	131 (15.9)	90 (60.8)	<0.001	205 (21.6)	16 (66.7)	<0.001
Treatments during hospitalization						
Antiplatelets, n (%)	768 (93.0)	124 (83.8)	<0.001	875 (92.1)	17 (70.8)	0.002
Thrombolysis, n (%)	74 (9.0)	24 (16.2)	0.007	94 (9.9)	4 (16.7)	0.292
Thrombectomy, n (%)	53 (6.4)	23 (15.5)	<0.001	70 (7.4)	6 (25.0)	0.008
Anticoagulants, n (%)	120 (14.5)	22 (14.9)	0.915	142 (14.9)	0 (0.0)	0.037
Lipid–lowering agents, n (%)	770 (93.2)	128 (86.5)	0.005	879 (92.5)	19 (79.2)	0.033
TOAST classification			<0.001			0.004
Large–artery atherosclerosis, n (%)	240 (29.1)	45 (30.4)		275 (28.9)	10 (41.7)	
Small–artery occlusion, n (%)	202 (24.5)	1 (0.7)		203 (21.4)	0 (0.0)	
Cardioembolic, n (%)	203 (24.6)	76 (51.4)		266 (28.0)	13 (54.2)	
Other etiology, n (%)	24 (2.9)	2 (1.4)		180 (18.9)	1 (4.2)	
Undetermined etiology, n (%)	157 (19.0)	24 (16.2)		26 (2.7)	0 (0.0)	

HT, hemorrhagic transformation; NHISS, National Institutes of Health Stroke Scale; SBP, systolic blood pressure; DBP, diastolic blood pressure; TG, triglyceride; TC, total cholesterol; LDL, low–density lipoprotein cholesterol; HDL, high–density lipoprotein cholesterol; MHR, monocyte to HDL cholesterol ratio.

**Figure 1:**
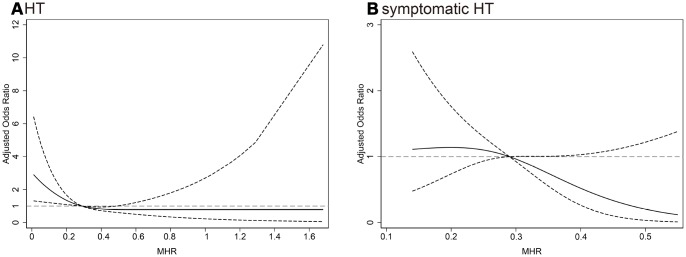
Multiple spline regression analyses were used to analyze the association between MHR and HT, (**A**) symptomatic HT (**B**) with three knots (at the 10th, 50th, 90th percentiles). Solid line indicates adjusted odds ratios, and dotted line indicates 95% confidence intervals. Reference of MHR was the midpoint (0.57). Odds ratios for HT were adjusted for age, sex, atrial fibrillation, drinking, NIHSS, systolic blood pressure, white blood cell, low-density lipoprotein cholesterol, large infarct size, antiplatelets, thrombolysis, thrombectomy, lipid-lowering agents after admission and TOAST classification, and for symptomatic HT were adjusted for NIHSS and large infarct size. MHR, monocyte to high-density lipoprotein cholesterol ratio; HT, hemorrhagic transformation; NIHSS, National Institutes of Health Stroke Scale score; TOAST, the Trial of ORG 10172 in Acute Stroke Treatment.

In multivariate logistic regression analysis, the MHR as a continuous variable was inversely associated with HT (odds ratio [OR] 0.22 per 1 standard deviation [SD] increase, 95% CI 0.06-0.78, *P* = 0.02). When MHR was classified into tertiles (Tertile 1: < 0.22; Tertile 2: 0.22-0.37; Tertile 3: > 0.37), the crude OR of HT in the lowest tertile was 1.85 (95% confidence interval [CI] 1.21-2.82, *P* = 0.004) compared with the highest tertile (Table 2). After adjustment for covariates, the lowest tertile group still had a significant higher risk of HT than highest tertile group (model 1: OR 1.75, 95% CI 1.06-2.89, *P* = 0.029; model 2: OR 1.81, 95% CI 1.08-3.01, *P* = 0.024) (Table 2). Similarly, the lowest tertile of MHR was associated with 3.82-fold increase in the odds of symptomatic HT (95% CI 1.04-14.00, *P* = 0.043) when compared with highest tertile after adjusting for NIHSS and large infarct size. Further analyses using restricted cubic spline regression showed that elevated MHR was associated with a decreased risk of HT and symptomatic HT (Figure 1).

**Table 2:** Multivariate logistic regression analysis between MHR and hemorrhagic transformation.

	**unadjusted**		**Model 1**		**Model 2**	
	**OR (95% CI)**	***P*-value**	**OR (95% CI)**	***P*-value**	**OR (95% CI)**	***P*-value**
MHR (Per 1 SD increase)	0.24 (0.08-0.71)	0.010	0.26 (0.08 - 0.91)	0.034	0.22 (0.06 - 0.78)	0.020
Tertiles of MHR						
Tertile 1 (< 0.22)	1.85 (1.21 - 2.82)	0.004	1.75 (1.06 - 2.89)	0.029	1.81 (1.08 - 3.01)	0.024
Tertile 2 (0.22 - 0.37)	0.98 (0.62 - 1.54)	0.919	0.92 (0.55 - 1.53)	0.755	0.85 (0.50 - 1.44)	0.547
Tertile 3 (> 0.37)	1		1		1	

Model 1: adjusted for age, sex, atrial fibrillation, drinking, National Institutes of Health Stroke Scale, systolic blood pressure, white blood cell, low-density lipoprotein cholesterol and large infarct size; Model 2: adjusted for covariates from Model 1 and further adjusted for antiplatelets, thrombolysis, thrombectomy, lipid-lowering agents after admission and the Trial of ORG 10172 in Acute Stroke Treatment classification. MHR, monocyte to high-density lipoprotein cholesterol ratio.

**Figure 2:**
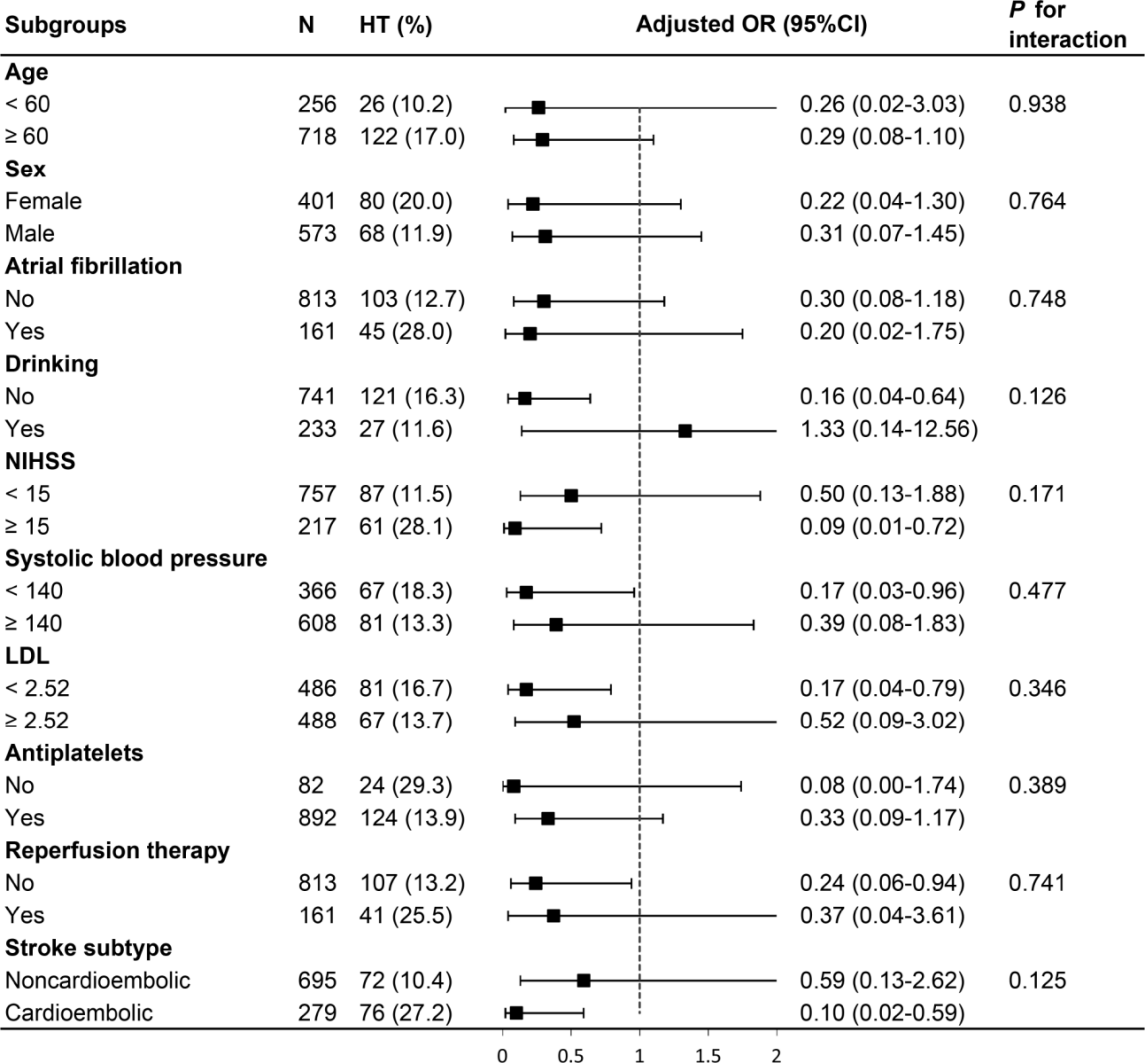
**Stratified logistic regression analysis to identify variables that modify the correlation between MHR and HT.** Above model adjusted for age, sex, atrial fibrillation, NIHSS, drinking, systolic blood pressure, LDL, antiplatelets, reperfusion therapy (thrombolysis/ thrombectomy) and stroke subtype. In each case, the model is not adjusted for the stratification variable. MHR, monocyte to high-density lipoprotein cholesterol ratio; HT, hemorrhagic transformation; NIHSS, National Institutes of Health Stroke Scale score; LDL, low-density lipoprotein cholesterol.

In subgroup analysis, the relationship between MHR and HT was not altered by age (< 60 versus ≥ 60), sex (male versus female), atrial fibrillation, stroke subtype (cardioembolism versus non-cardioembolism), alcohol consumption, baseline NIHSS score (< 15 versus ≥ 15), baseline systolic blood pressure (< 140 versus ≥ 140), low-density lipoprotein cholesterol (< 2.52 versus ≥ 2.52), antiplatelets used after admission, and reperfusion therapy (thrombolysis/thrombectomy). No significant interaction was observed (*P* for interaction > 0.05 for all; Figure 2).

## DISCUSSION

We provided preliminary evidence about the relationship between MHR and HT in patients with acute ischemic stroke. Our study indicated that low MHR was independently associated with increased risk of HT and symptomatic HT, after adjustment for important confounders. The results remained consistent across subgroups stratified by age, sex, atrial fibrillation, stroke subtype, stroke severity, and those with or without antiplatelet and reperfusion therapy.

In the present study, the incidence of HT was 15.2%, which fell within the broad range of 7.5% - 49.5% reported in previous studies [2–5]. The low rate of symptomatic HT (2.5%) could be explained by the inclusion of a non-selected cohort of consecutive patients regardless of whether thrombolysis/thrombectomy is performed. Nevertheless, we confirmed the established predictive factors of HT that are evident in studies focusing on reperfusion therapies. These factors included age, atrial fibrillation, greater NIHSS score, large infarct size, increased WBC counts and decreased low-density lipoprotein cholesterol [18–20]. Moreover, when stratified by reperfusion treatment, we observed no statistically significant difference in the association of MHR with HT, suggesting generalizability of our results.

The MHR value, defined as the division of monocyte count to HDL value, is a novel inflammatory indicator and is positively correlated with poor outcomes in several cardiovascular diseases [15, 21, 22]. Few studies have previously looked at the role of MHR in stroke. Findings from a population-based Chinese cohort indicated elevated MHR levels were significantly associated with higher prevalence of ischemic stroke [23]. In addition, MHR has been reported to be an independent predictor of 30-day mortality in patients with acute ischemic stroke [24]. Furthermore, in patients with acute intracerebral hemorrhage, higher MHR was shown to be associated with increased risk of death or disability [25]. However, studies specifically focused on the association between MHR and HT in humans have not been found. In the present study, we demonstrated that low admission MHR was associated with increased risk of HT and symptomatic HT after ischemic stroke.

The mechanism by which MHR exerts a protective effect of HT is not clear. Several possible explanations have been proposed. The first explanation is related to the dual role of monocytes/macrophagocytes. Distinct subpopulations of monocytes/macrophages may critically determine the outcome of lesion-associated inflammation [26]. Many investigations have indicated that monocytes/macrophagocytes play an important role in the pro-inflammatory process, in which M1 macrophagocyte is a detrimental factor [27–29]. Indeed, apart from proinflammatory effects, monocytes/macrophagocytes have noticeable anti-inflammatory effects, mainly by M2 macrophagocytes. In animal experiments, M2 macrophagocytes release anti-inflammatory mediators such as transforming growth factor - β1 to prevent hemorrhagic infarct transformation [11]. Additionally, M2 macrophagocytes may enhance repair of brain endothelial cells and promote angiogenesis [30], which could maintain integrity of blood brain barrier, thus preventing HT eventually [18]. The second explanation is in regarding to HDL cholesterol. HDL plays a vital role in anti- atherosclerosis, anti-inflammation and antioxidant stress, [31], which may reduce breakdown of blood brain barrier. However, existing studies have indicated paradoxical results that higher HDL levels on admission were associated with increased risk of HT [12, 13], which was in agreement with our results in univariate analyses. Additionally, some evidence suggests that HDL could inhibit adhesion of monocytes to endothelium [32], possibly leading to attenuated protective effect of M2 macrophagocytes. Despite these results, the exact roles of monocytes/macrophagocytes and HDL involved in HT are still unclear. Future research should aim to explore underlying mechanism in animal and human studies.

Although the present study provides new insights about influence of MHR on the risk of HT, it has some limitations. First, the present study was a retrospective analysis based on a single center stroke registry database. Therefore, these data may not reflect the whole Chinese population or other ethnic populations. Second, MHR was not dynamically measured, it was unknown whether temporal changes of MHR are related to HT. Third, the number of patients with HT or symptomatic HT was relatively low, which may limit the statistical power. Fourth, some patients were excluded for presenting HT on admission, without repeated imaging or no data on monocyte and HDL, which may lead to selection bias and limit the generalizability of our findings. Finally, residual confounding cannot be excluded as severe infection, nutrition status, and other complications after stroke possibly affect the MHR values. For these reasons, further studies are needed to verify our findings and to determine the underlying causes.

## CONCLUSION

Lower MHR was independently associated with increased risks of HT, especially symptomatic HT after acute ischemic stroke. MHR may serve as a promising marker for identifying patients with higher risk of HT and help clinicians choose appropriate treatment to avoid potential bleeding risks. Further preclinical and clinical studies are warranted to clarify the complex effects of the immune system and inflammation in the pathophysiology of ischemic stroke and HT.

## MATERIALS AND METHODS

### Subjects

Data in this study were obtained from the Chengdu Stroke Registry, which consecutively recruited ischemic stroke patients admitted to the Department of Neurology, West China Hospital. Details of the registry has been described previously [33]. Patients aged ≥ 18 years admitted within 24 h of stroke onset between January 2016 to September 2018 were considered eligible. Diagnosis of ischemic stroke was made according to the World Health Organization criteria [34] and confirmed by CT or MRI. Exclusion criteria were: (1) occurrence of HT at hospital admission; (2) without a second CT or MRI scan; (3) lack of data on serum monocyte or HDL within 24 h of admission. In addition, 100 healthy controls (64.9 ± 6.9 years; 60 males) without ischemic stroke were included from our hospital during physical check-ups. This study was approved by the Biomedical Research Ethics Committee of West China Hospital, Sichuan University, and conformed to local and international ethical criteria. Informed consent was obtained from patients or their next of kin.

### Data collection

We used a standardized form to collect demographics, vascular risk factors (hypertension, diabetes mellitus, hyperlipidemia, atrial fibrillation, current smoking and drinking), previous medication use (antiplatelets, lipid-lowering agents and anticoagulants), baseline blood pressure, and treatments during hospitalization. Trained neurologists assessed the baseline stroke severity using NIHSS and determined the probable stroke etiology based on TOAST classification [35]. Venous blood samples were collected within 24 h of hospital admission and delivered to the laboratory department of our hospital. WBC counts and their differentials were measured by automated cell counters with standard techniques. Lipid profiles and other biochemical indices were analyzed enzymatically via automatic biochemical analyzer. The MHR was calculated by dividing the monocyte count and HDL level. All patients underwent a head CT on admission, followed by scheduled MRI within 7 days after admission or repeated CT when clinical worsening occurred. MRI were performed using 3.0 Tesla Siemens Trio MR scanner. Details of the MRI parameters have been described in our previous study [36]. Large infarct size was defined as parenchymal hypoattenuation involving more than 1/3 of the middle cerebral artery territory.

### Definition of HT

HT is referring to the hemorrhage within the infarct territory or parenchyma hemorrhage outside the infarct zone that was detected later on follow up CT or MRI, but not on initial CT [37]. Symptomatic HT is defined as hemorrhage that is associated with any decline in neurologic status [38]. Two trained researchers blind to clinical data rated HT on imaging. Disagreement was resolved by consensus or consulting a third neurologist.

### Statistical analysis

Continuous variables were presented as Mean ± SD or median (IQR) and were analyzed with Student’s t test and Mann–Whitney U test. Categorical variables were represented as frequencies (percentage) and compared using the chi-squared test or Fisher’s exact test. Logistic regression was performed to estimate the association between MHR and HT. Variables with *P* < 0.1 in the univariate analysis or established predictors of HT were further adjusted in multivariate logistic regression model. Unadjusted and adjusted OR and 95% CI were calculated. In addition, we used logistic regression model with restricted cubic splines to explore the pattern and magnitude of the association between MHR (continuous variable) and HT with three knots (at the 10^th^, 50^th^, 90^th^ percentiles), adjusting for covariates. Stratified logistic regression models were used to perform subgroup analyses. Interactions between MHR and stratified factors were tested by the likelihood ratio test. All statistical analyses were performed using Stata 15.0 (StataCorp LP, College Station, TX, USA), and R version 3.4.3 (R Foundation for Statistical Computing, Vienna, Austria). A two-tailed *P* < 0.05 was considered statistically significant.
